# Primary health care and nutrition

**DOI:** 10.2471/BLT.20.251413

**Published:** 2020-05-28

**Authors:** Christian Kraef, Benjamin Wood, Peter von Philipsborn, Sudhvir Singh, Stefan Swartling Peterson, Per Kallestrup

**Affiliations:** a Heidelberg Institute of Global Health, University of Heidelberg, Im Neuenheimer Feld 672, 69120 Heidelberg, Germany.; b School of Health and Social Development, Deakin University, Victoria, Australia.; c Institute for Medical Informatics, Biometry & Epidemiology, Ludwig-Maximilians University, Munich, Germany.; d Faculty of Medical and Health Sciences, University of Auckland, New Zealand.; e Health Section, United Nations Children's Fund, New York, United States of America.; f Centre for Global Health, Department of Public Health, Aarhus University, Denmark.

## Abstract

Globally, dietary factors are responsible for about one in five deaths. In many low- and middle-income countries different forms of malnutrition (including obesity and undernutrition) can co-exist within the same population. This double burden of malnutrition is placing a disproportional strain on health systems, slowing progress towards universal health coverage (UHC). Poor nutrition also impedes the growth of local economies, ultimately affecting the global economy. In this article, we argue that comprehensive primary health care should be used as a platform to address the double burden of malnutrition. We use a conceptual framework based on human rights and the Astana Declaration on primary health care to examine existing recommendations and propose guidance on how policy-makers and providers of community-oriented primary health care can strengthen the role of nutrition within the UHC agenda. Specifically, we propose four thematic areas for action: (i) bridging narratives and strengthening links between the primary health care and the nutrition agenda with nutrition as a human rights issue; (ii) encouraging primary health-care providers to support local multisectoral action on nutrition; (iii) empowering communities and patients to address unhealthy diets; and (iv) ensuring the delivery of high-quality promotive, preventive, curative and rehabilitative nutrition interventions. For each theme we summarize the available strategies, policies and interventions that can be used by primary health-care providers and policy-makers to strengthen nutrition in primary health care and thus the UHC agenda.

## Résumé

Environ un décès sur cinq dans le monde est dû à des facteurs alimentaires. Dans de nombreux pays à faible et moyen revenu, différentes formes de malnutrition (y compris l'obésité et la dénutrition) peuvent coexister au sein d'une même population. Ce double fardeau de malnutrition exerce une pression démesurée sur les systèmes de santé, ralentissant la progression vers une couverture maladie universelle (CMU). Une mauvaise alimentation entrave également la croissance des économies locales, ce qui en fin de compte affecte l'économie mondiale. Dans cet article, nous estimons qu'il est impératif d'utiliser une approche globale des soins de santé primaires comme plateforme pour s'attaquer au double fardeau de la malnutrition. Nous avons employé un cadre conceptuel fondé sur les droits humains et la Déclaration d'Astana sur les soins de santé primaires. D'une part pour examiner les recommandations existantes, et d'autre part pour fournir un éclairage sur la manière dont les législateurs et les prestataires de soins de santé primaires, implantés au niveau communautaire, peuvent renforcer le rôle de la nutrition dans le programme de CMU. Nous proposons plus exactement quatre champs d'action : (i) aligner les discours et consolider les liens entre les soins de santé primaires et le programme de nutrition, en intégrant ce dernier dans la thématique des droits humains; (ii) encourager les prestataires de soins de santé primaires à soutenir les initiatives locales multisectorielles portant sur la nutrition; (iii) donner aux patients et aux collectivités le pouvoir de lutter contre l'alimentation déséquilibrée; et enfin, (iv) assurer la mise en œuvre d'interventions de qualité pour la promotion, la prévention, le traitement et la réhabilitation en matière de nutrition. Pour chaque champ d'action, nous résumons les stratégies, politiques et interventions à la disposition des législateurs et prestataires de soins de santé primaires pour renforcer le rôle de la nutrition dans les soins de santé primaires et, par conséquent, le programme de CMU.

## Resumen

Los factores alimentarios son responsables de aproximadamente una de cada cinco muertes en todo el mundo. Diferentes tipos de malnutrición (incluidas la obesidad y la desnutrición) pueden coexistir en la misma población de muchos países de ingresos bajos y medios. Esta doble carga de la malnutrición está ejerciendo una presión desproporcionada sobre los sistemas sanitarios, lo que ralentiza los progresos hacia la cobertura sanitaria universal (CSU). Además, la mala nutrición dificulta el crecimiento de las economías locales, lo que en última instancia afecta a la economía global. En este artículo, se argumenta que la atención primaria de salud integral se debería utilizar como plataforma para abordar la doble carga de la malnutrición. Se utiliza un marco conceptual basado en los derechos humanos y en la Declaración de Astaná sobre la atención primaria de salud para analizar las recomendaciones existentes y proponer directrices sobre cómo los responsables de formular las políticas y los proveedores de atención primaria de salud orientada a la comunidad pueden fortalecer la función de la nutrición dentro del programa de la CSU. En concreto, se proponen cuatro áreas temáticas de acción: (i) narrativas de vinculación y fortalecimiento de los vínculos entre la atención primaria de salud y el programa de nutrición en donde la nutrición sea una cuestión de derechos humanos; (ii) alentar a los proveedores de atención primaria de salud a que apoyen la medida multisectorial local sobre la nutrición; (iii) potenciar a las comunidades y a los pacientes para tratar las dietas poco saludables; y (iv) garantizar la realización de intervenciones de nutrición de alta calidad de tipo promocional, preventivo, curativo y de rehabilitación. Para cada tema se resumen las estrategias, políticas e intervenciones disponibles que los proveedores de atención primaria de salud y los responsables de formular las políticas pueden utilizar para fortalecer la nutrición en la atención primaria de salud y, por consiguiente, el programa de la CSU.

## ملخص

على المستوى العالمي، تعتبر العوامل الغذائية مسؤولة تقريباً عن حالة واحدة من كل خمس وفيات. يمكن أن تتعايش أشكال مختلفة من سوء التغذية في العديد من الدول منخفضة الدخل ومتوسطة الدخل (بما يشمل السمنة وفقر التغذية) ضمن نفس السكان. يضع هذا العبء المزدوج لسوء التغذية ضغطاً غير متناسب على النظم الصحية، مما يبطئ من التقدم نحو التغطية الصحية الشاملة (UHC). كما تعوّق التغذية السيئة نمو النظم الاقتصادية المحلية، مما يؤثر في النهاية على النظام الاقتصاد العالمي. نحن نقرر في هذه المقالة أنه يجب الاستعانة بالرعاية الصحية الأولية الشاملة كمنصة للتعامل مع العبء المزدوج لسوء التغذية. نحن نستخدم إطارًا مفاهيمي يعتمد على حقوق الإنسان وإعلان الأستانة بشأن الرعاية الصحية الأولية لفحص التوصيات القائمة، واقتراح إرشادات حول كيف يمكن لواضعي السياسة ومقدمي الرعاية الصحية المجتمعية الأولية، تعزيز دور التغذية في جدول أعمال التغطية الصحية الشاملة. نحن نقترح على وجه التحديد أربعة مجالات مواضيعية للعمل: (1) ترابط الإجراءات وتعزيز الروابط بين الرعاية الصحية الأولية، وجدول أعمال التغذية مع التغذية، كقضية من قضايا حقوق الإنسان؛ و(2) تشجيع مقدمي الرعاية الصحية الأولية على دعم إجراءات الدعم المحلي متعدد القطاعات بشأن التغذية؛ و(3) تمكين المجتمعات والمرضى للتعامل مع الأنظمة الغذائية غير الصحية؛ و(4) ضمان تقديم تدخلات تغذوية تأهيلية وعلاجية وقائية ومشجعة عالية الجودة. بالنسبة لكل موضوع، نحن نقوم بتلخيص الاستراتيجيات والسياسات والتدخلات المتاحة، والتي يمكن استخدامها بواسطة مقدمي الرعاية الصحية الأولية، وواضعي السياسات لتعزيز التغذية في الرعاية الصحية الأولية، وبالتالي جدول أعمال التغطية الصحية الشاملة.

## 摘要

在全球范围内，大约五分之一的死亡与饮食因素相关。在许多中低收入水平的国家内，同一人群中可能同时存在不同形式的营养不良（包括肥胖症和缺乏营养）。营养不良的双重负担给卫生系统造成了太大的压力，减缓了全民健康覆盖 (UHC) 的进程。营养不良也阻碍了当地经济的增长，最终影响了全球经济。在本文中，我们认为应将全面的初级卫生保健用作解决营养不良双重负担的平台。我们采用基于人权的概念框架以及关于初级卫生保健的《阿斯塔纳宣言》来审查现有的建议，并且针对政策制定者和面向社区的初级卫生保健提供者可以如何在全民健康覆盖 (UHC) 议程中强调营养的作用提供指导。具体而言，我们提出了四个主题行动领域：(i) 以营养作为人权问题，衔接叙述并加强初级卫生保健和营养议程之间的联系；(ii) 鼓励初级卫生保健提供者支持当地有关营养的多部门行动；(iii) 使社区和患者有能力解决不健康的饮食问题；以及 (iv) 确保提供高质量的促进性、预防性、治疗性和康复性营养干预措施。我们针对每个主题总结了可供初级卫生保健提供者和决策者在初级卫生保健以及全民健康覆盖 (UHC) 议程中采用的加强营养策略、政策和干预措施。

## Резюме

Каждая пятая смерть в мире связана с факторами питания. Во многих странах с низким и средним уровнем дохода в рамках одной и той же популяции могут встречаться различные формы нарушения питания, включая ожирение и недоедание. Такое двойное бремя неполноценного питания налагает несоразмерную нагрузку на системы здравоохранения, замедляя прогресс в обеспечении всеобщего охвата услугами здравоохранения (ВОУЗ). Неудовлетворительное питание также подрывает рост местной экономики и в конечном счете оказывает влияние на глобальную экономику. В этой статье авторы приводят доводы в пользу того, что комплексную первичную медико-санитарная помощь следует использовать в качестве платформы для решения проблемы двойного бремени неполноценного питания. Авторы используют концептуальную схему, основанную на правах человека и Астанинской декларации по вопросам первичной медико-санитарной помощи, для изучения существующих рекомендаций и составления руководящих указаний, призванных помочь ответственным за принятие решений и поставщикам социально ориентированной первичной медико-санитарной помощи в усилении роли питания для обеспечения ВОУЗ. В частности, предлагаются четыре направления деятельности: (i) избавление от укоренившихся стереотипов и укрепление связей между первичной медико-санитарной помощью и вопросами питания, в которых питание рассматривается как часть прав человека; (ii) поощрение поставщиков первичной медико-санитарной помощи к оказанию поддержки местным многосекторальным мероприятиям в области питания; (iii) расширение прав и возможностей общин и пациентов для решения вопросов, связанных с неполноценным питанием; (iv) проведение высококачественных рекламных, профилактических, лечебных и реабилитационных мероприятий в сфере питания. Для каждой темы авторы приводят сводку доступных стратегий, политик и вмешательств, которые могут использоваться поставщиками первичной медико-санитарной помощи и лицами, ответственными за принятие решений, для улучшения питания в системе первичной медико-санитарной помощи и достижения поставленных целей в области ВОУЗ.

## Introduction

About one in five deaths globally are attributable to poor diets, making dietary factors responsible for 11 million deaths annually, more than any other risk factors covered by the Global Burden of Disease study.^1^ More than two billion adults are overweight or obese; both are known risk factors for numerous noncommunicable diseases, notably cardiovascular disease and type 2 diabetes.^2^ Almost two-thirds of infants between 6 months and 2 years old do not receive an adequate diet, putting them at-risk of the short- and long-term health effects of poor nutirition.^3^

In many low- and middle-income countries different forms of malnutrition, including undernutrition and obesity, can co-exist within the same population. More than half of deaths in children younger than 5 years are due to diet-related risk factors, particularly undernutrition, while 41 million children in the same age group are obese.^4^^,^^5^ This evidence of a double burden of malnutrition prompted a series of papers in 2019 calling for double-duty action to address malnutrition in all its forms.^6^ The distribution of the burden of these diet-related risk factors and diseases is highly unequal, both within and between countries.^7^^,^^8^ Malnutrition drives disease and stunts economic growth, costing the global economy approximately 3.5 trillion United States dollars (US$) per year.^9^ As the prevalence of malnutrition and diet-related noncommunicable diseases grow, these economic impacts increase. An even stronger economic case exists when considering the impact of the inefficiencies and environmental impacts of the food systems that produce such unhealthy diets, in addition to the direct health-related costs themselves. A conservative estimate of the potential economic benefits to society from addressing the current such hidden costs of food systems sums to US$ 5.7 trillion annually by 2030, increasing to US$ 10.5 trillion annually by 2050.^10^

We argue that tackling this double burden of malnutrition is an urgent global health and development challenge. We provide an overview of similarities and links across the nutrition, primary health care and universal health coverage (UHC) agendas, presenting past and present debates on this issue. We use a conceptual framework based on human rights and the Astana Declaration of 2018 on revitalizing primary health care^11^ to examine existing recommendations and propose guidance for policy-makers on how community-oriented primary health care can strengthen the role of nutrition within the UHC agenda. 

## Nutrition and sustainable development

The challenge of malnutrition is intrinsically linked to the United Nations 2030 agenda for sustainable development.^12^ Several of the sustainable development goals (SDGs) are directly related to nutrition: zero hunger (SDG 2), good health and well-being (SDG 3) and responsible consumption and production (SDG 12). Nutrition can be considered an outcome of the global food system, the complex network of food-related activities including the production, processing, transport, marketing and consumption of food. The global food system is among the largest drivers of global environmental change, including climate change, biodiversity and habitat loss, and land degradation.^13^ Thus, from a planetary health perspective, nutrition is also closely linked to other SDGs: access to water and sanitation for all (SDG 6), climate action (SDG 13) and life on land (SDG 15). A fundamental target for SDG 3 is UHC, defined as ensuring that all people have access to needed health services, including prevention, promotion, treatment, rehabilitation and palliation, of sufficient quality to be effective, while also ensuring that the use of these services does not expose the user to financial hardship.^14^

Nutrition and UHC are linked in several ways. First, progress towards UHC is threatened by the growing burden of malnutrition and noncommunicable diseases, which are placing a disproportional strain on health systems and are threatening to reverse progress towards UHC. Second, UHC can serve as a framework for programmatic action for improved nutrition in the population. The World Health Organization (WHO) has described primary health care as the programmatic engine of UHC.^15^ Primary health care is defined as an overall approach, which encompasses multisectoral policy and action to address the broader determinants of health; empowering individuals, families and communities; and meeting people’s essential health needs throughout their lives.^16^ In this context primary care is a subset of primary health care and refers to essential, first-contact care provided in a community setting.^16^

Comprehensive primary health care is well-positioned to serve as a link between the nutrition and UHC agendas. First, the nutrition and primary health care agendas are similar in concept. Both require a human-rights based framework, multisectoral action, community involvement and a life-course based delivery of evidence-based preventive and curative health care integrated with public health services.^11^^,^^17^ Second, the primary health-care sector is the place where primary and secondary prevention can be scaled up, disadvantaged population groups reached and treatments for diet and nutrition-related diseases delivered most cost–efficiently.^18^ Finally, primary health-care providers witness on a daily basis the burden of poor nutrition and unhealthy diets, such as malnutrition-related child mortality or the adverse outcomes of obesity. Primary health-care providers’ role at the frontline of the health-care system makes them natural advocates for improvements in the production, processing, transport, marketing and consumption of food.^19^ Two examples are the minimum unit pricing policy for alcohol in Scotland that was advocated by primary health-care providers^20^ and advocacy for climate-responsible health-care systems by Health Care Without Harm, an international nongovernmental organization.^21^

The contribution of primary health care to improved nutrition was explicitly outlined in the Alma-Ata Declaration of 1978.^22^^,^^23^ In a speech in 1982, Halfdan Mahler, the then Director-General of the WHO and a key architect of the Declaration, argued that primary health care improves nutrition when countries have: (i) explicit nutritional objectives; (ii) sustained and adequate levels of nutritional care components; (iii) integrated monitoring and evaluation mechanisms for nutrition-related outcomes; and (iv) community involvement.^22^ Furthermore, he emphasized intersectoral action for nutrition and health and the role of health professionals in promoting intersectoral policy-making. More recently, the relationship between UHC and nutrition was highlighted by WHO in a policy brief on nutrition in UHC,^24^ which made the point that UHC cannot be achieved without ensuring equitable access to quality nutrition services. The document highlighted the role of primary health care, in combination with secondary and tertiary care, as a platform for addressing the determinants of unhealthy diets both within and beyond the health system.

## Framework for action on nutrition

Four interconnected thematic areas, based on human rights and the Astana Declaration on primary health care, can be used as a framework for action on nutrition. In this article we summarize the available strategies, policies and interventions for each area. The first thematic area explains how primary health care can bridge the gap between systems- and individual-level thinking in nutrition. Discussing nutrition in the context of primary health care strengthens the notion of nutrition as a human right of immediate, tangible relevance in people’s lives. The second thematic area originates in the view that primary health-care providers are in a unique position to identify and contribute to multisectoral solutions on the local level. We examine how primary health-care providers can make a difference by improving governance mechanisms, working towards public awareness and empowerment and making local food production healthier, and by influencing and shaping local policy and regulations. The third thematic area focuses on community empowerment to identify reasonable ways to involve the community in addressing dietary patterns and their local determinants. Such activities include increased nutrition literacy, community classes and adequate sensitization and training of health professionals. In the fourth thematic area we outline some the most important promotive, preventive, curative and rehabilitative nutrition interventions, and how to provide them in an equitable and non-stigmatizing way.

### Nutrition as a human right

Both diet and nutrition need to become a central part of the medical and scientific discourse in primary health-care institutions and local health administrations. This approach has the potential to foster community-led advocacy to improve existing food systems and to contribute to more community-oriented ways in which knowledge is gathered, understandings are reached and priorities are set.^25^ Making clear that nutrition is an inseparable part of comprehensive primary health care brings nutrition within the human rights-based foundation of the Alma-Ata and Astana Declarations and strengthens equity considerations, and the right to safe and nutritious food. The power of using a human rights-centred approach in addressing complex global health issues is reflected in the global response to the epidemic of human immunodeficiency virus infection. In 2019, scientists and health policy-makers and providers called for the development of international guidelines on human rights, healthy diets and sustainable food systems.^26^

Sustainable change needs a compelling narrative.^27^ The focus of discourse about action on nutrition in global health has shifted to social, political and commercial determinants of nutritional health, and how to change the global food system to modify these determinants.^28^^,^^29^ This shift contrasts with the traditional biomedical model that emphasizes individual responsibility and the need for medical interventions. However, neither of these two viewpoints alone represents the experiences and possibilities of individuals and communities. The emphasis on social, political and commercial determinants of health is far removed from the individual. Conversely, the biomedical model risks placing too much responsibility and blame on the individual, who is often deprived of the choice or possibility to act. Community-oriented primary health care can bridge this gap between systems-level and individual-level thinking. The links between both models become clearer when looked at from a comprehensive primary health-care perspective. This perspective recognizes the principles of multisectoral action, community involvement and integrated delivery of promotive, preventive, curative and rehabilitative services.^18^ To alter the narrative to integrate both macrolevel and individual determinants, the nutrition agenda needs to be considered in the global, national and local policy planning processes, where health systems and health-related development policies are discussed. Global fora, for example the World Health Assembly, United Nations General Assembly and initiatives such as the Global Nutrition Summit (scheduled in Japan in 2020) can be platforms to work towards concrete financial and political commitments intended to strengthen the role of nutrition in primary health care.^24^

### Local actions for better nutrition

A unique strength of primary health-care providers is their ability to identify and contribute to multisectoral solutions on the local level that are adapted to community needs.^30^ The advantage of local knowledge is well-demonstrated by primary health-care providers’ use of social prescribing to link patients to a range of local, non-clinical services across multiple sectors in a community (for example, cookery classes, volunteering and exercise groups).^31^ In the context of nutrition, primary health-care providers, by being embedded at the local level, can act to shape food environments. Food environments can be defined as the physical, economic, political and sociocultural context by which consumers engage with the food system to make their decisions about acquiring, preparing and consuming food.^32^

There are several of existing frameworks and examples of action on public health nutrition. In 2009, the Brazilian government published the Matrix for Food and Nutrition Actions in Primary Health Care. The matrix is a framework for teams of health professionals from diverse areas of knowledge to systematize and organize the food and nutrition actions and nutritional care components in primary health care.^33^ In 2019, the EAT-Lancet Commission on Food in the Anthropocene suggested some crucial strategies to change food systems.^28^ Specifically, the report called for complex systemic interactions that take place across food systems to be addressed at both a global scale and at scales adapted to local realities and needs.^28^ The United Nations Environmental Programme outlined four action steps in a collaborative framework for food systems transformation: (i) identifying a group of food systems champions; (ii) conducting a holistic system assessment; (iii) initiating a multistakeholder process for dialogue and action; and (iv) strengthening institutional capacity for long-term food systems governance.^34^ Actions (i) to (iii) of this approach are close to the multisectoral nature of comprehensive primary health care and can be directly supported by primary health-care providers at the local level (Box 1). For action (iv), primary health care can play an important role by holding policy-makers and politicians accountable based on their immediate experience of policy impact, high public trust and roles as patient advocates.

Box 1 **Contributions of primary health-care providers to local multisectoral action for better food systems**

**Strong and coordinated governance**

- Advocate an evidence-based, health-in-all policies approach at municipal, regional and national level.
- Initiate and participate in local multistakeholder partnerships and coordinating mechanisms (e.g. with municipalities, the agricultural and marine industries, food retailers and other stakeholders), where appropriate and feasible.
- Promote community participation as a central element for policy coherence and the success of local government initiatives.

**Public awareness and empowerment**

- Raise public awareness on nutrition and diet-related diseases.
- Mobilize health professionals and their professional organizations for improving nutrition care, research and policy.
- Be at the frontline of local and national early warning, alert and response networks relating to food safety issues and nutrition emergencies.
- Engage and support local communities to develop and lead healthy eating and physical activity initiatives, including a focus on capacity-building and training of community staff.
- Contribute to educational curricula on nutrition, including meal planning, food shopping and budgeting, and food storage and preparation.

**Healthy food production**

• Contribute expertise on healthy diets in non-health related local policy settings (e.g. zoning regulations for urban horticulture).

• Coordinate and create links between the work of community health workers and agricultural extension agents.

• Advocate for and support elements of community-supported agriculture as a dietary and health improvement strategy.

• Contribute to shifts in consumer demand through campaigns and education on healthy diets.

**Local policy and regulation**

- Provide locally contextualized, evidence-based guidance on food and nutrition to policy-makers.
- Advocate for evidence-based policies (e.g. a local tax on non-nutritious foods and sugary drinks) and support the creation of healthy food environments in the retail and food service sector (e.g. through nudging strategies).
- Advocate for reduced promotion of unhealthy food and beverages in the media (e.g. television and radio), at public and community events and publicly managed settings (e.g. public transport, local recreational facilities) and through digital platforms.
- Promote regulatory measures to further support mothers to breastfeed (e.g. changes to maternity leave, facilities and time for breastfeeding in the workplace).
- Advocate for changes to the food procured and provided in schools, public sector canteens and hospitals, and advise the food-service industries.
- Consider promoting fortification of condiments and staple foods with vitamins and minerals in line with relevant evidence-based guidelines.
- Advocate for schools and early child-care settings to address unhealthy diets through integrated interventions and support them where needed, including the following:
- systematic surveillance of weight and nutrition status;
- promotion of physical activity; and
- advocating for appropriately trained professionals to be placed in educational facilities, highlighting the importance of education on healthy lifestyles from an early age.
- Advocate for a built environment which supports healthy food choices and physical activity, with a specific focus on children (e.g. through urban planning and zoning regulations).

Sources: Swinburn et al., 2019.^4^ International Panel of Experts on Sustainable Food Systems, 2017.^25^ Vasquez et al., 2017.^35^

Altering the macrolevel determinants of people’s diets is also needed, through mechanisms such as improved trade, agricultural and taxation policies, as well as better regulation of the powerful private-sector forces, such as multinational corporations active in the global food system.^36^ Advocacy by primary health-care providers, including nutritionists, nurses and doctors, and fostered in primary health-care institutions, can be an important driver for commitment to macro-level policy change. Such an approach has been demonstrated by the climate and health movement.^37^

### Empowering communities and people

Primary health-care providers are usually well placed to understand the local dietary context and identify reasonable ways to involve the community in addressing dietary patterns and their local determinants of health. Healthy diets are about much more than the science of nutritional components and how to optimize production and consumption of the right nutrients. After all, it is generally dietary patterns rather than individual food products that affect people’s health. Socioeconomic factors such as culture, religion, gender, geographical location and employment status are important determinants of human diets. In many cultures, eating is a communal activity with social interaction that means much more than just providing fuel for the human body. Policies and interventions that recognize factors that are embedded within the local context are likely to be sustainable and effective. An example is the Brazilian government’s dietary guidelines which “are designed to be sustainable personally, culturally, socially, economically and environmentally.”^38^

Considering the relevant local factors could also help to address power imbalances between health systems and the people they serve. Health systems can be made more person-centred and equity-oriented, such as by prioritizing those with greater need by allocating greater resources to the vulnerable. This approach is also relevant to the delivery of health and nutrition literacy and education programmes in an equitable manner that ideally takes into account the individual agency required of each community member for sustainable change.

Empowerment of communities and individuals to address unhealthy diets through primary health-care structures involves the provision of information, and the strengthening of nutrition literacy, the ability to obtain, read, understand and use nutrition information. Effective distribution channels include primary health-care providers in health-care facilities, public institutions, such as schools, and public awareness campaigns in the mass media. One example is community-based nutrition education, including cooking skills, which is a worthwhile approach in many settings, for instance as a part of community health schemes that also provide training to caterers and food providers in public service facilities.^39^ These approaches can be supported by coordination across different sectors and by the employment of health outreach teams in schools, at social events or public food markets. To do this, primary health-care providers should ensure that clinics employ appropriately trained professionals to offer classes and consultations. By ensuring that local health-care facilities are exemplars in the provision of healthy food options, primary health-care providers can advocate for large procurers of food, such as municipal canteens, to follow suit. Positive examples are the comprehensive food policy in New York city, United States of America, or the People’s Restaurants initiative in Belo Horizonte city, Brazil.^40^^,^^41^ New York’s food policy aims to improve the municipality’s food governance by increasing local and sustainable food procurement, reducing the consumption of meat and setting chronic disease-related health standards. In Belo Horizonte the initiative provides cheap, healthy, safe and accessible meals made from local produce for about 1 United States dollar per a meal (half-price for beneficiaries of the government’s social welfare programme and free to homeless people).^42^

### High-quality nutrition interventions

Primary health-care providers can play a key role in delivering promotive, preventive, curative and rehabilitative nutritional interventions as part of comprehensive care. Health professionals in primary care are well-placed to assess patients’ diets, screen for dietary risk factors, to diagnose obesity and other diet-related diseases early, and to take appropriate action. Comprehensive primary health-care services are also in a good position to ensure health equity by reaching all groups of people, including those who are marginalized, especially when health care is free at the point of care. A greater degree of individual agency is often required for an individual-level intervention to be effective. Interventions are therefore more likely to preferentially benefit people of higher socioeconomic status compared with those with more limited social and economic resources.^43^ Accordingly, interventions focused only on education and behaviour change could reinforce existing inequities.^44^ Primary health-care services can rebalance this equity consideration by prioritizing resources and attention on the most vulnerable individuals.^45^

A major limitation in many primary health-care systems is the lack of training of health professionals for dietary assessments, dietary counselling and the prevention and treatment of malnutrition, and its related diseases.^46^^,^^47^ All health-care professionals, including nutritionists, nurses, midwives and community health workers, need to be able to practice to the full scope of their skill-set to deliver nutrition interventions.^48^ Training of health-care providers should address bias against patients with obesity and teach behaviour change strategies, the ability to work within multidisciplinary teams and the nature of food systems.^49^ One way to prioritize nutrition appropriately is by providing adequate remuneration for promotive, preventive, curative and rehabilitative care. Data collection and evaluation of a wide range of diet and nutrition-relevant metrics, including the diet-related burden of disease and health-system capacity indicators, need to become core elements of comprehensive primary health-care systems. A range of different data sources, such as standardized dietary surveys, and morbidity and mortality data, will be needed.

The potential for digital health systems to deliver nutrition counselling and promotive, as well as preventive actions in primary health care, should be realized and scaled systematically based on available evidence.^50^ The evidence is still inconclusive for many interventions, such as mobile phone-based applications that focus on weight-loss education and improved behaviour.^51^ There is, however, growing evidence in some areas, such as the effectiveness of mobile applications to improve breastfeeding and postnatal nutrition in low- and middle-income countries.^52^ Box 2 provides an overview of the most important nutritional interventions that could be delivered within primary health care.^61^

Box 2 **Role of primary health care in promotive, preventive, curative and rehabilitative interventions for better nutrition**

- Provide advice on healthy diets and appropriate nutrition, as well as other lifestyle factors (e.g. physical activity, sedentary behaviour, alcohol and tobacco use).
- Carry out growth assessment, counselling and referral.
- Provide treatment for malnutrition-related disorders, including wasting and stunting.
- Administer micronutrient supplements, including vitamin A and zinc, when appropriate.
- Conduct early identification of obesity in citizens, particularly children.
- Counsel patients with obesity on weight loss or refer to specialist care, consistent with evidence-based national clinical guidelines and the local context.
- Provide pre-conception, antenatal and postpartum nutrition guidance and support for healthy pregnancy, including iron and folic acid supplements.
- Promote, protect and support breastfeeding; ensure all health-care settings adopt best-practice breastfeeding policies and practices.
- Ensure patients with comorbidities (e.g. tuberculosis and human immunodeficiency virus infection) receive appropriate nutritional advice.
- Identify elderly people with or at risk of malnutrition and provide appropriate preventive and curative care in line with national clinical guidelines.
- Integrate dietary assessments and counselling in the educational curricula for primary health-care professionals.

Sources: World Health Organization (WHO), 2019.^24^ United Nations Children’s Fund (UNICEF), 2005.^53^ WHO, 2017.^54^ WHO, 2018.^55^ WHO, 2018.^56^ Moore et al., 2000.^57^ UNICEF, 2019.^58^ Wadden et al., 20181.^59^ Brown et al., 2019.^60^

## Conclusion

We have focused on four thematic areas for action in primary health care to strengthen the role of nutrition within the UHC agenda: (i) bridging narratives and strengthening links between the primary health care and nutrition agendas, with nutrition as a human rights issue; (ii) encouraging primary health-care providers to support local multisectoral action on nutrition; (iii) empowering communities and patients to address unhealthy diets; and (iv) ensuring the delivery of high-quality promotive, preventive, curative and rehabilitative nutrition interventions. We believe this framework of available strategies, policies and interventions can serve to address the human suffering caused by the growing burden of malnutrition and noncommunicable diseases, and to pre-empt the disproportional strain on health systems that threatens to jeopardize progress towards UHC.
